# Curcumin induces apoptosis and inhibits proliferation in infantile hemangioma endothelial cells via downregulation of MCL-1 and HIF-1α

**DOI:** 10.1097/MD.0000000000009562

**Published:** 2018-02-16

**Authors:** Suhui Lou, Yanfang Wang, Zujiang Yu, Kelei Guan, Quancheng Kan

**Affiliations:** a Department of Pharmacology; b Department of Infectious Diseases, The First Affiliated Hospital of Zhengzhou University, Zhengzhou, Henan Province, China.

**Keywords:** apoptosis, curcumin, endothelial cells, infantile hemangioma

## Abstract

**Background:**

Curcumin has been used as an alternative medicine for the treatment of infantile hemangiomas (IHs); however, the mechanism underlying the effectiveness of curcumin in IHs remains largely unclear.

**Methods:**

In this study, we isolated primary human hemangioma endothelial cells (HemECs) from fresh surgical specimens of 3 patients. We treated HemECs by curcumin and investigated the alterations in proliferative and apoptotic signaling pathways with cell counting kit-8, flow cytometry, western blotting, immunofluorescence, and real-time polymerase chain reaction.

**Results and Conclusion:**

We found that curcumin potently inhibited proliferation in HemECs, achieving low-micromolar IC50 (the half maximal inhibitory concentration) value. We also observed that treatment with curcumin induced apoptosis in HemECs, as evidenced by positively Annexin-V-FITC staining, caspase-3 activation, and cleavage of poly(adenosine diphosphate-ribose) polymerase (PARP) in the treated cells. Moreover, we showed that curcumin suppressed the expression of antiapoptotic protein myeloid cell leukemia-1 (MCL-1), hypoxia-inducible factor 1α (HIF-1α), and vascular endothelial growth factor (VEGF).

Altogether, our study suggests that the effectiveness of curcumin in IHs may be associated with its potent antiproliferative and apoptotic activities in HemECs.

## 1 Introduction

Infantile hemangioma (IH) is the most common benign soft tissue tumors in infants.^[1,2]^ IH is pathologically characterized by rapid proliferation of endothelial cells at the initial phase followed by a phase of slow involution.^[3]^ By completely removing the tumors or destroying the abnormal vasculature, localized therapies, such as surgical incision, cryosurgery, laser, radiation, and hardener treatments, can achieve satisfactory efficacy in most patients with small IHs.^[4]^ But, for patients with lesions causing disfiguring, blocking airway, or lesions in the internal organs, systemic treatments are often warranted. Corticosteroids, propranolol, and anticancer drugs vincristine and bleomycin are mainstay of treatments for IHs.^[5,6]^ Nonetheless, unsatisfactory efficacy and/or severe side effects may preclude patients from using these medicines, highlighting the need to explore alternative modalities for this disease.^[7,8]^

Curcumin is a bioactive component extracted from *rhizome of Curcuma longa Linn*, which has been broadly used for the management of various human diseases in India and China for centuries.^[9]^ Numerous studies suggest that curcumin possesses a variety of biological activities such as anti-inflammatory, antioxidant, and antitumor effects.^[10]^ Interestingly, previous studies showed that curcumin also could inhibit the growth of hemangiomas and several other types of vascular diseases.^[11,12]^ For instance, Hassell and Roanh^[12]^ reported that dietary supplement of a large amount of curcumin resulted in the complete remission of a life-threatening infantile liver hemangioma in a 6-month-old infant. These findings demonstrated that curcumin has the potential to be used for the treatment of IHs. However, the mechanism underlying the effectiveness of curcumin in IHs remains to be clearly elucidated.

## 2 Materials and methods

### 2.1 Tissue samples

IH tissues were obtained from freshly surgical specimens of 3 young male patients (2, 2.3 and 3 years old). The diagnosis of IH for these patients was made based upon clinical examination and computed tomography with and without contrast in the Department of Pediatric Surgery in the First Affiliate Hospital of Zhengzhou University. This study was approved by the Ethics Committee of the First Affiliate Hospital of Zhengzhou University. Both patients’ parents were consent informed.

### 2.2 Cells culture and identification

Endothelial cells were isolated from the proliferating-phase specimens as described previously.^[13]^ Briefly, the hemangioma samples were minced into small pieces using a scalpel and digested with collagenase I (Sigma, St. Louis, MO). The HemECs were purified from primary cultures using a flow cytometer (BD Bioscience, San Jose, CA) to separate cells. The HemECs were plated in RPMI1640 supplemented with 10% heat-inactivated fetal bovine serum (FBS, Gibco, Solarbio, Beijing), streptomycin (100 μg/mL, Solarbio, Beijing), and penicillin (100 U/mL, Solarbio, Beijing). The cells were cultured at a humidified atmosphere of 37°C, with 5% CO_2_, and stained by specific markers for hemangioma of factor VIII, CD31, and CD34. Staining was performed according to standard techniques as previously reported.^[14]^ Human umbilical vein endothelial cells (HUVECs) were obtained from MT Biological Technology Co (Shanghai, China) and cultured as described previously.^[15]^

### 2.3 Cell viability CCK-8 assay

Freshly isolated HemECs seeded at 3000 cell/well in a 96-well plate were cultured overnight, then were treated with a serial dilution of curcumin (Sigma-Aldrich, Shanghai, China) at concentrations of 0, 6.25, 12.5, 25, 50, and 100 mM in RPMI1640 containing 10% FBS for 48 hours. To measure cell viability, water-soluble tetrazolium-1 cell counting kit-8 (CCK-8, Dojindo, Japan) solution was added to 96-well plates at a final concentration of 10% and incubated for 4 hours, and the absorbance was measured at 450 nm.

### 2.4 Flow cytometry apoptosis assay

HemECs seeded in 60-mm plates were treated with curcumin at 25 μM or dimethyl sulfoxide (DMSO) control for 48 hours. Cells were harvested with pancreatic enzymes without ethylenediaminetetraacetic acid. Apoptotic cells were determined by Annexin V-FITC/propidium iodide (PI) staining using the Annexin V-FITC Apoptosis Detection Kit (Roche Diagnostics, Shanghai, China) according to the manufacturer's instructions. Data were analyzed with Cell Quest software (BD Biosciences, Sparks, MD).

### 2.5 Morphology analysis using transmission electron microscopy

HemECs seeded in 60-mm plates were treated with curcumin at 25 μM or DMSO control for 48 hours. Transmission electron microscopy was performed following the manufacturer's instructions. Briefly, the cells were harvested and fixed in 2.5% glutaraldehyde, embedded over night at 37°C in propylene oxide and epoxy resin, and cut into ultrathin sections (1–2 μm). Sections were double-stained with uranyl acetate and lead citrate and observed via electron microscopy at a magnification of 60,000 × (H-7650, HITACHI Co, Ltd, Tokyo, Japan).

### 2.6 Immunofluorescence analysis

Cell suspension density was adjusted to (4–2) × 10^4^/mL and then grown in chamber slides (Thermo Fisher Scientific, Shanghai, China). After static adherence, HemECs were exposed to 0 and 25 μM curcumin for 24 hours. Then cells were washed in phosphate buffer solution (PBS) for 3 times and fixed with 4% paraformaldehyde for 10 minutes. Permeabilization was performed by treatment with 0.1% Triton X-100 for 5 minutes. Endogenous catalase was neutralized by 0.3% H_2_O_2_. The cells were incubated with 10% goat serum in PBS to prevent nonspecific reaction and incubated with primary antibodies (β-actin, BOSTER BM0627, mouse polyclonal, 1:100; HIF-1α, Abcam 23426, rabbit polyclonal, 1:75; VEGF, BBI AB60788a, rabbit polyclonal, 1:300; Ang-2, BBI, AB20217a, rabbit polyclonal, 1:500) at 4°C overnight. After washes for 3 times, the cells were incubated with secondary antibody labeled fluorescence for 30 minutes, follow by 4’,6-diamidino-2-phenylindole (DAPI) staining. The cells were then examined and photographed using a fluorescence microscope (ZEISS, Germany).

### 2.7 Real-time polymerase chain reaction analysis

HemECs seeded in 60-mm plates were treated with curcumin at 25 μM or DMSO control for 24 hours. The total RNA was isolated using TRIZOL Reagent (Invitrogen, Carlsbad, CA) and reverse transcripted to complementary DNA (cDNA) according to the RevetAidTMFirst Strand cDNA Synthesis Kit (Fermentas, Thermo Fisher Scientific, Shanghai, China) instructions. The gene expression was detected using SYBR Premix Ex TapTM II (TaKaRa) and *glyceraldehyde-3-phosphate dehydrogenase* (*gapdh*) gene was served as the internal reference. All primers were synthesized by TaKaRa (Dalian, China) as shown in Table 1. Real-time polymerase chain reaction (RT-PCR) was performed in triplicate utilizing the StepOnePlusTM Real-Time PCR System (Applied Biosystems, Beijing, China). The results were normalized to *gapdh* level and the triplicate results were averaged for each sample. Data were analyzed using the comparative *C*_*t*_ method (2−ΔΔ*C*_*t*_).

**Table 1:**
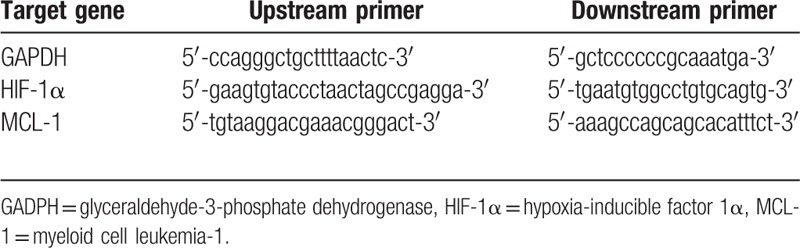
Primer sequence of target genes.

### 2.8 Western blotting analysis

The cells were lysed in radioimmunoprecipitation assay buffer supplemented with protease inhibitor cocktail (Roche, Shanghai, China). Equal amounts of protein were loaded and separated by sodium dodecyl sulfate polyacrylamide gel electrophoresis, transferred to polyvinylidene fluoride membranes, and detected by immunoblotting using the enhanced chemiluminescence Prime Western Blotting Detection Kit (GE Healthcare). GAPDH was used as a loading control. The primary antibodies for caspase-3 (9661), MCL-1 (94296), and GAPDH (5174) were all purchased from Cell Signaling Technology (Shanghai, China).

### 2.9 Caspase-3 activity assay

The caspase-3 activity assay in the cell lysates was determined with the caspase-3 activity assay kit (5723) from Cell Signaling Technology (Shanghai, China).

### 2.10 Statistical analysis

The data were expressed as mean ± SD for a representative experiment performed in triplicate. Statistical significance was determined by a Student's *t*-test using GraphPad Prism (GraphPad Software Inc). Differences were considered to be statistically significant if *P* <.05.

## 3 Results

### 3.1 HemECs display biological characteristics of endothelial cells

**Figure 1:**
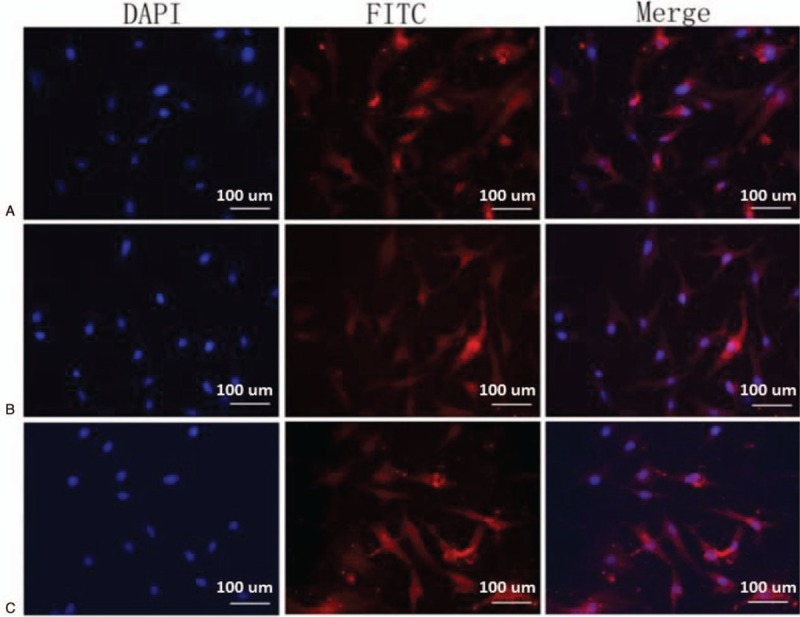
Cells isolated from the proliferating-phase specimens of IHs display characterization of HemECs. Endothelial cells isolated from the proliferating-phase specimens of IHs were grown in chamber slides and fixed with cold methanol. The expressions of factor VIII (A), anti-CD31 (B), anti-CD34 (C) were examined with immunofluorescence analysis and specific antibodies. The cells were also stained with DAPI as blue background signal. Representative photographs from 3 independent experiments are shown. CD = cluster of differentiation, DAPI = 4′,6-diamidino-2-phenylindole, IH = infantile hemangiomas.

To characterize the cells isolated from IH tissue, we seeded the cells in chamber slides and allowed the cells to attach and grow for 24 hours. The expressions of CD31, CD34, and factor VIII in the cells were examined by immunofluorescent staining. We found that almost all cells expressed all 3 molecules with high intensities (Fig. 1). Since these 3 cell-surface molecules were used as biomarkers for identification of endothelial cells, these results suggest that most cells isolated from IH tissues were HemECs and were suitable for further investigation.

### 3.2 Curcumin inhibits cell proliferation of HemECs in a dose-dependent manner

**Figure 2:**
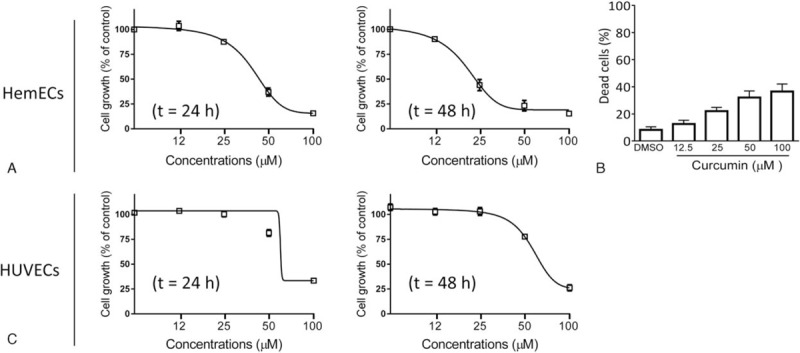
Curcumin potently inhibits the proliferation of HemECs. (A) HemECs were treated with curcumin at concentrations of 6.25, 12.5, 25, 50, and 100 μm for 24, and 48 hours. Cell proliferation was determined using the CCK-8 assay. The data are presented by mean ± standard deviation of 3 independent experiments. (B) HemECs were treated with curcumin at concentrations of 12.5, 25, 50, and 100 μm for 48 hours. Cell death was determined by the trypan blue exclusion assay. (C) HUVECs were treated with curcumin at concentrations of 6.25, 12.5, 25, 50, and 100 μm for 24 and 48 hours. Cell proliferation was determined using the CCK-8 assay. The data are presented by mean ± standard deviation of 3 independent experiments. CCK-8 = cell counting kit-8, HemECs = hemangioma endothelial cells, HUVECs = human umbilical vein endothelial cells.

To examine the inhibitory effect of curcumin on the proliferation of HemECs, we treated the cells by serial dilutions (6.25–100 μM) of the drug for 24 and 48 hours and examined the cell viability with the CCK-8 assay. We found that treatment with curcumin dose-dependently inhibited cell proliferation (Fig. 2A). The IC50 values of curcumin were 38 and 31 μM for 24 and 48 hours, respectively. We noted that curcumin at 25 μM inhibited cell proliferation by 56% at 48 hours, and we thus used this concentration for a mechanistic study. In the trypan blue assay, we found that curcumin treatment for 48 hours led to cell death in a dose-dependent manner (Fig. 2B).

HUVECs are commonly used as relative normal endothelial cells in biology research.^[15]^ We next treated HUVECs by serial dilutions of curcumin and examined the activity of the drug in these cells (Fig. 2C). The results showed that curcumin also inhibited cell viability in HUVECs. However, the activity of curcumin was much weaker in these cells as compared to that in HemECs, with IC50 values 73 and 69 μM at 24 and 48 hours time points, respectively.

### 3.3 Curcumin suppresses the expression of HIF-1α and VEGF in HemECs

**Figure 3:**
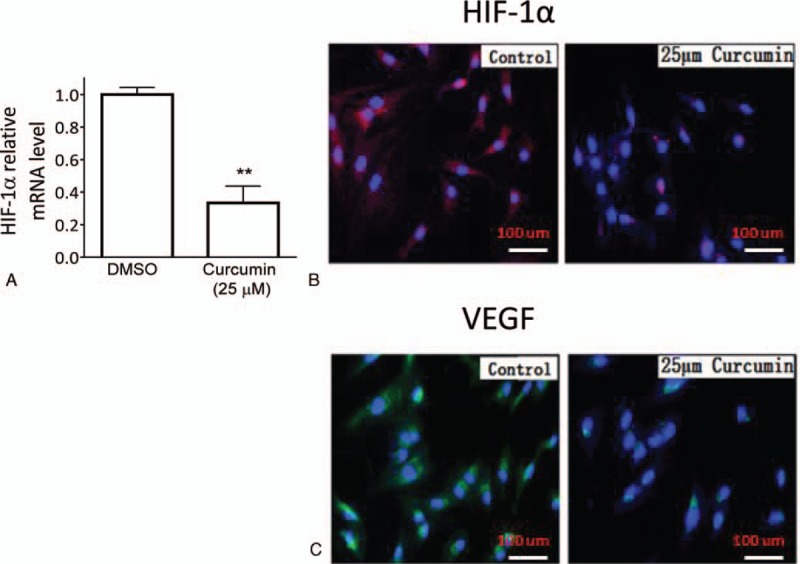
Curcumin induces downregulation of HIF-1α and VEGF in HemECs. HemECs were treated with 25 μM curcumin or DMSO for 48 hours. (A) The mRNA level of HIF-1α in treated cells was examined by the RT-PCR assay. The expressions of (B) HIF-1α and (C) VEGF were examined with immunofluorescence analysis and specific antibodies. The cells were also stained with DAPI as blue background signal. Representative photographs from 3 independent experiments are shown. DAPI = 4’,6-diamidino-2-phenylindole, DMSO = dimethyl sulfoxide, HemECs = hemangioma endothelial cells, HIF-1α = hypoxia-inducible factor 1α, RT-PCR = real-time polymerase chain reaction, VEGF = vascular endothelial growth factor.

It was reported that curcumin was able to downregulate HIF-1α and MCL-1 in HUVECs.^[16]^ We thus next investigated whether curcumin could have similar effect on the expression level of HIF-1α in HemECs. Our data showed that treatment with 25 μM curcumin for 48 hours significantly reduced the mRNA level of HIF-1α (Fig. 3A). We further performed immunofluorescent staining to visualize the effect of curcumin on the protein expression of HIF-1α and VEGF, one of HIF-1α key downstream targets in HemECs. The results showed that the immunofluorescent signaling of both HIF-1α and VEGF from cells treated by curcumin treatment was much weaker than cells treated by DMSO control, suggesting the inhibition of HIF-1α and VEGF expression by curcumin (Fig. 3B and C). Since VEGF is a key factor for the growth of endothelial cells, these data therefore suggest that the inhibition of HIF-1α-VEGF axis may contribute to the antiproliferative activity of curcumin.

### 3.4 Curcumin suppresses the expression of MCL-1 and triggers apoptosis signaling in HemECs

**Figure 4:**
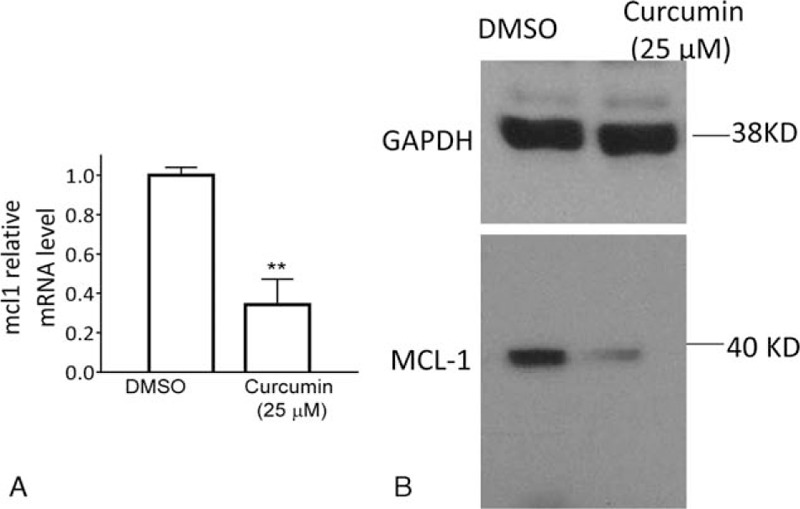
Curcumin inhibits MCL-1 in HemECs. HemECs were treated with 25 μM curcumin or DMSO for 48 hours. (A) The mRNA level of MCL-1 in treated cells was examined by the RT-PCR assay. (B) The protein level of MCL-1 in treated cells was examined by western blotting analysis. GAPDH was used as a loading control. DMSO = dimethyl sulfoxide, GAPDH = glyceraldehyde-3-phosphate dehydrogenase, HemECs = hemangioma endothelial cells, MCL-1 = myeloid cell leukemia-1, RT-PCR = real-time polymerase chain reaction.

The RT-PCR assay also showed that treatment with 25 μM curcumin for 48 hours substantially reduced the mRNA level of MCL-1 (Fig. 4A). Western blotting analysis demonstrated that inhibition of gene expression translated to a marked decrease in the expression level of the MCL-1 protein in HemECs (Fig. 4B).

**Figure 5:**
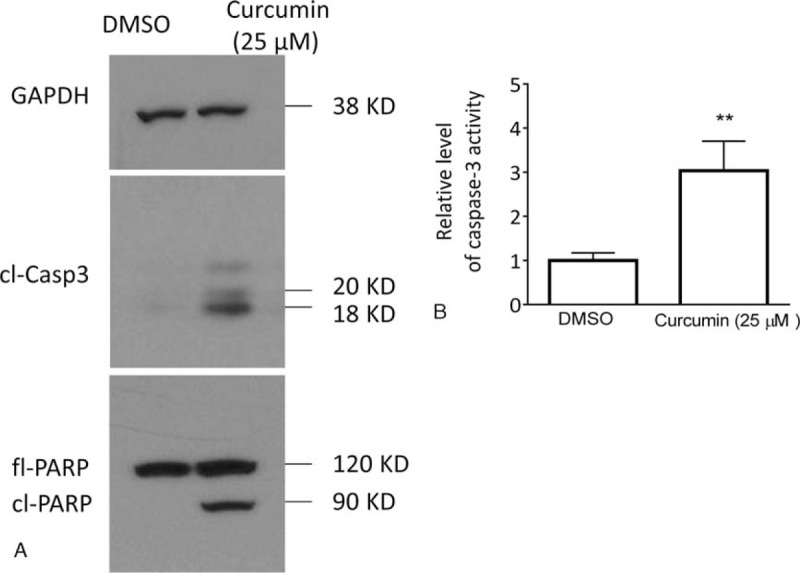
Curcumin triggers apoptosis signaling in HemECs. (A) HemECs were treated with 25 μM curcumin or DMSO for 48 hours. The protein levels of cl-casp3 and PARP (full length-PARP and cleaved PARP) were examined by western blotting. GAPDH was used as a loading control. (B) HemECs were treated with 25 μM curcumin or DMSO for 48 hours. The level of caspase-3 activity was examined with Caspase-3 Colorimetric Protease Assay Kit according to manufactory protocol. The data are presented by mean ± standard deviation of 3 independent experiments. Cl-casp3 = cl-caspase-3, DMSO = dimethyl sulfoxide, GAPDH = glyceraldehyde-3-phosphate dehydrogenase, HemECs = hemangioma endothelial cells, PARP = poly(adenosine diphosphate ribose) polymerase.

Since MCL-1 is a critical antiapoptotic Bcl-2 family member, we next investigated whether the inhibition of MCL-1 by curcumin would trigger activation of apoptosis signaling in HemECs. Western blotting analysis showed that treatment by 25 μM curcumin for 48 hours induced apparent accumulation of cleaved caspase-3 and cleavage of full-length poly(adenosine diphosphate ribose) polymerase (PARP) (Fig. 5A). Fluorescent biochemical assay showed that curcumin increased the caspase-3 activity by 3 folds as compared to DMSO control (Fig. 5B).

### 3.5 Curcumin induces apoptosis in HemECs

**Figure 6:**
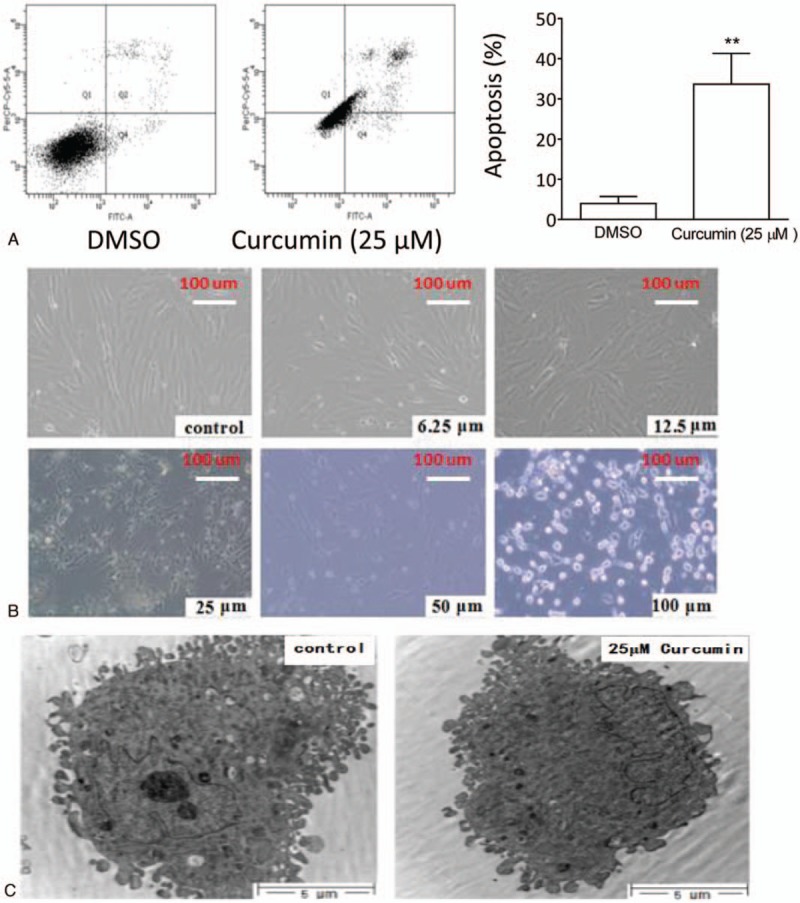
Curcumin induced apoptosis of HemECs. (A). HemECs were treated with 25 μM curcumin or DMSO for 48 hours. Treated cells stained with Annexin-V/PI were examined by flow cytometry. (Left panels) Representative plots of apoptosis from 3 independent experiments are shown. (Right panels) The percentages of apoptosis are plotted. The data are presented by mean ± standard deviation of 3 independent experiments. ∗∗*P* <.01. (B) HemECs were treated with different concentrations of curcumin or DMSO for 48 hours. Morphological alteration of treated cells were examined under light microscopy and photographed. Representative graphs for each treatment from 3 independent experiments are shown. (C) HemECs were treated with 25 μM curcumin or DMSO for 48 hours. Ultrastructure alteration of treated cells was examined with transmission electron microscopic examination. DMSO = dimethyl sulfoxide, HemECs = hemangioma endothelial cells, PI = propidium iodide.

Because caspase-3 activation and PARP cleavage are hallmarks for apoptotic signaling,^[17]^ the above-mentioned findings stimulated us to investigate the apoptotic activity of curcumin in HemECs. We treated the cells with curcumin at 25 μM for 48 hours, then stained the treated cells with Annexin-V-FITC and PI and determined the percentage of apoptotic cells by flow cytometry. We found that the treatment with curcumin at this concentration led to a significant increase in the percentage of HemEC apoptosis compared with the control treatment (33.7 ± 3.6% vs 3.9 ± 0.2%; *P* <.05) (Fig. 6A).

Under light microscopy, we observed that HemECs treated by curcumin for 48 hours showed obvious apoptosis-like morphological alterations. Curcumin at low concentrations caused the cells detached from the plates and from other cells, and at high concentrations caused cells shrunk and floating in the medium (Fig. 6B).

Transmission electron microscopic examination was carried to examine the alterations in HemECs after treatment by curcumin. The results showed that the treated cells displayed ultrastructural apoptotic morphological characteristics, such as nuclear body formation with condensed chromatin, nuclear fragmentation, nuclear change of chromatin clumping, as well as membrane complex fragmentation (Fig. 6C).

Altogether, these results demonstrate that curcumin potently induces apoptosis in HemECs.

## 4 Discussion

Curcumin, a natural polyphenol compound from the perennial herb *C longa*, has been proved to have beneficial effects in treatment of malignant and benign tumors, inflammation and many other conditions.^[9,10]^ It has been observed that treatment with curcumin lead to the remission of a liver HI.^[1,2]^ However, there was a controversy over whether the cure of the HI was caused by the treatment of curcumin or just was the result of spontaneous regression.^[12]^ To provide some insights for this issue, we carried out this study with freshly isolated HemECs. We found that curcumin displayed potent antiproliferative activity in HemECs. Since abnormal overgrowth of HemECs is the pathological basis for IHs, our results therefore present a rationale for using curcumin in management of HIs.

HIF-1α is known to be a key regulator in hypoxia-induced angiogenesis, which is a major proangiogenic factor in many hypoxic solid tumors,^[18]^ and also is associated with the growth of hemangiomas.^[19]^ We found that curcumin significantly repressed the expression of HIF-1α, as well as VEGF, a key downstream effector of HIF-1α pathway in HemECs. It has been reported that curcumin inhibits cell proliferation by inhibiting HIF-1α in human pituitary adenoma cells.^[20]^ Our findings thus suggest that inhibition of HIF-1α-VEGF axis may also contribute to the antiproliferative activity of curcumin in HemECs. Moreover, it has been reported that HIF-1α regulates MCL-1 transcription in both malignant and normal cells.^[21]^ This suggests that the inhibition of HIF-1α may also contribute to the suppression of MCL-1.

Our data showed that curcumin treatment led to typical apoptotic morphological alterations, Annexin-V-positive staining, as well as activation of caspase-3 in HemECs. These suggest that induction of HemECs apoptosis may be involved in the anti-IH activity by curcumin. Moreover, our study indicates that curcumin displays a certain extent of selectivity in targeting HemECs over HUVECs. We assume that this selectivity may be attributed to the abnormal cellular structures and fast dividing nature of HemECs. Apoptosis resistance has been believed to be an important characteristic of the IH endothelial cells during the proliferation phase.^[22]^ Our findings thus indicate that in order to maximize treatment effects, curcumin should be applied for patients with early stage of IHs.

Apoptosis is regulated by Bcl-2 family proteins which are characterized by the presence of Bcl-2 homology (BH) domains in their structure.^[23]^ Bcl-2 family proteins are classified into proapoptosis and antiapoptosis subfamily members.^[24]^ A number of previous studies have reported that curcumin inhibits MCL-1 in cancer cells.^[25,26]^ We here showed that curcumin also effectively repressed the expression of MCL-1 in HemECs. These evidences suggest that the inhibitory ability on the expression of MCL-1 by curcumin is not restricted in malignant cancer cells. Given that MCL-1 belongs to the subfamily of the antiapoptosis Bcl-2 protein, which is a key negative regulator for apoptosis signaling,^[27]^ we propose that MCL-1 inhibition is responsible, at least partially for the apoptosis induction by curcumin in HemECs.

Overall, our study demonstrates that curcumin displays potent antiproliferative and apoptosis activities in HemECs, which may be through the inhibition of MCL-1 and HIF-1α. Our findings provide a rational basis for clinical application of curcumin in the treatment of IHs and also provide insight for the understanding of the cellular and molecular mechanisms of curcumin in the treatment of IHs.
